# Within-person stability of lipoprotein(a) concentration

**DOI:** 10.1093/eurheartj/ehae652

**Published:** 2024-10-08

**Authors:** Jonas Ghouse, Gustav Ahlberg, Søren Albertsen Rand, Morten Salling Olesen, Bjarni Vilhjalmsson, Stefan Stender, Henning Bundgaard

**Affiliations:** Department of Cardiology, Rigshospitalet, Copenhagen University Hospital, Blegdamsvej 9, 2100 Copenhagen, Denmark; Cardiac Genetics Group, Department of Biomedical Sciences, University of Copenhagen, Blegdamsvej 3B, 2200 Copenhagen, Denmark; Department of Clinical Immunology, Aarhus University Hospital, Palle Juul-Jensens Blvd. 99, 8200 Aarhus, Denmark; Department of Cardiology, Rigshospitalet, Copenhagen University Hospital, Blegdamsvej 9, 2100 Copenhagen, Denmark; Cardiac Genetics Group, Department of Biomedical Sciences, University of Copenhagen, Blegdamsvej 3B, 2200 Copenhagen, Denmark; Department of Cardiology, Rigshospitalet, Copenhagen University Hospital, Blegdamsvej 9, 2100 Copenhagen, Denmark; Cardiac Genetics Group, Department of Biomedical Sciences, University of Copenhagen, Blegdamsvej 3B, 2200 Copenhagen, Denmark; Department of Cardiology, Rigshospitalet, Copenhagen University Hospital, Blegdamsvej 9, 2100 Copenhagen, Denmark; Cardiac Genetics Group, Department of Biomedical Sciences, University of Copenhagen, Blegdamsvej 3B, 2200 Copenhagen, Denmark; Department of Clinical Immunology, Aarhus University Hospital, Palle Juul-Jensens Blvd. 99, 8200 Aarhus, Denmark; Department of Clinical Biochemistry, Rigshospitalet, Copenhagen University Hospital, Copenhagen, Denmark; Department of Clinical Medicine, University of Copenhagen, Copenhagen, Denmark; Department of Cardiology, Rigshospitalet, Copenhagen University Hospital, Blegdamsvej 9, 2100 Copenhagen, Denmark; Department of Clinical Medicine, University of Copenhagen, Copenhagen, Denmark

**Keywords:** Lipoprotein(a), Measurement, Variability, Stability

## Introduction

Lipoprotein(a) (Lp(a)) is a causal risk factor for cardiovascular disease.^1^ Given the strong genetic influence on Lp(a) concentrations, a single measurement throughout the course of life has been suggested.^2^ Here, we evaluate the temporal stability of Lp(a) as a function of baseline Lp(a) levels.

## Methods

The UK Biobank (UKB) is an observational study that enrolled over 500 000 individuals aged 40–69 between 2006 and 2010. In this analysis, we focused on 12 202 participants with baseline and follow-up Lp(a) measurements (data field 30 790). Lipoprotein(a) was measured in nmol/L at enrolment using an immunoturbidimetric method on the Beckman Coulter AU5800 platform (Randox Biosciences, UK), which is isoform insensitive.^3^ Only measurements within the assay’s analytical range (3.8–189 nmol/L) were reported in this data field. Individuals were categorized based on Lp(a) concentrations at baseline into low (<75 nmol/L), intermediate (75–125 nmol/L), and high (126–189 nmol/L) levels.^2^ We used Spearman correlation and intraclass correlation coefficient (ICC) to assess the correlation between baseline and follow-up Lp(a). We used Bland–Altman plots to visually display measurement variability. We calculated the percentage of individuals who moved from one risk category to another across successive measurements. We calculated the percentage of individuals that experienced an absolute change of ≥25 nmol/L according to different baseline reference levels. We also investigated the temporal stability of measurements flagged as being outside the assay’s analytical range (>189 nmol/L, data field 30 796). For these measurements, the actual value is not provided, but is instead flagged as ‘Not reported at assay (too high)’. Using these data, we examined the proportion of individuals who either remained outside the analytical range or moved into one of the previously defined categories. The research was conducted under UKB application 43247.

## Results

**Figure 1:**
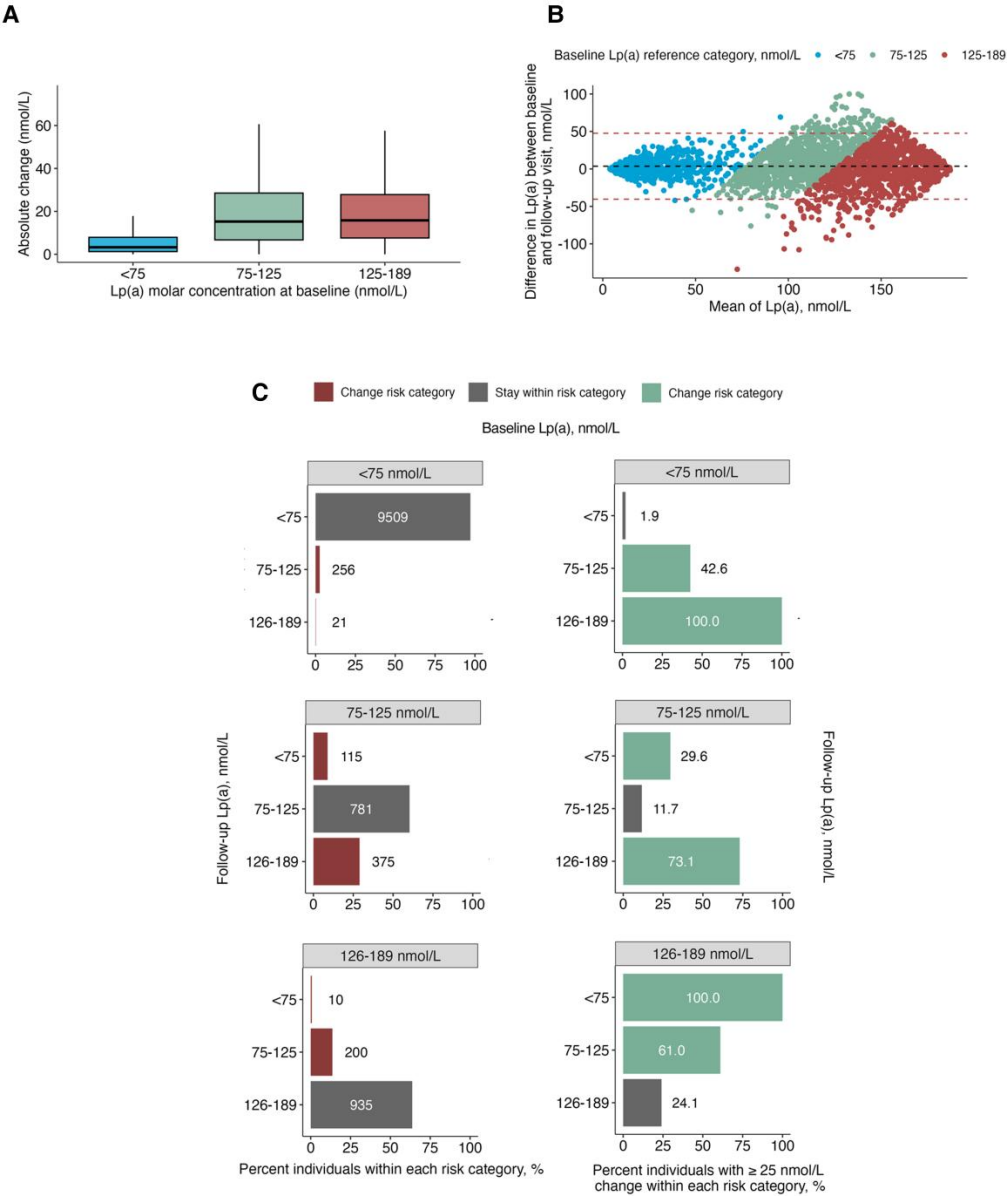
(*A*) Box plot displays the absolute change in nmol/L between the baseline and follow-up measurement according to baseline reference category. Within each box plot, the horizontal lines indicate the median, the top and bottom of each box indicate the interquartile range, and the whiskers indicate the maximum and minimum values within each grouping no further than 1.5 × interquartile range from the hinge. (*B*) The Bland–Altman plot shows the differences between the baseline and repeat measurement of lipoprotein(a). For ease of interpretation, a random sample of 1000 individuals was selected. Each dot is coloured according to individual baseline risk category (low/intermediate/high). The black dashed line represents the mean difference, and the red dashed lines represent the 95% confidence intervals. (*C*) Left-hand column of bar plots shows the fraction of individuals changing risk categories from baseline to follow-up measurement according to different baseline lipoprotein(a) strata. Right-hand column of bar plots shows the fraction of individuals with an absolute change of ≥25 nmol/L within each baseline and follow-up lipoprotein(a) strata

Median Lp(a) was 21.0 nmol/L (interquartile range [IQR] 9.8–57.1) at baseline, increasing to 22.4 nmol/L (IQR 10.4–60.7) at follow-up. The median time between successive measurements was 4.4 years (IQR 3.7–4.95). The overall correlation was strong between the two measurements [Spearman *r =* 0.94, *P* < .0001; ICC 0.974, 95% confidence interval (CI) 0.973–0.975], but varied by baseline Lp(a) levels (*Figure 1A*). For individuals with Lp(a) < 75 nmol/L, we found a strong correlation with the follow-up measurement (Spearman *r* = 0.90, *P* < .0001; ICC 0.923, 95% CI 0.920–0.926). However, with higher baseline levels, the correlation deteriorated, with *r* of 0.50 (*P* < .0001; ICC 0.564, 95% CI 0.513–0.609) for levels between 75 and 125 nmol/L and *r* of 0.39 (*P* < .0001; ICC 0.505, 95% CI 0.444–0.559) for higher reference values (125–189 nmol/L). This can also be observed in the Bland–Altman plot (*Figure 1B*), which shows increasing variability with higher baseline Lp(a). Next, we assessed the proportion of individuals who experienced significant absolute changes in Lp(a) across successive measurements, stratified by different reference levels. While only 3.2% of individuals with lower Lp(a) levels (<75 nmol/L) had changes of ≥25 nmol/L, significant changes were observed for 31.4% of individuals with intermediate baseline Lp(a) levels and 31.2% of individuals with high baseline Lp(a) levels. Out of 1271 (10.4% of total study sample, *Figure 1C*) individuals with an intermediate Lp(a) level at baseline, 375 (29.5%) moved to a higher Lp(a) risk category at the follow-up visit, while 115 (9.0%) were reclassified as low risk. Among the 1145 (9.4% of total study sample, *Figure 1C*) individuals initially classified with high Lp(a) levels, 200 (17.5%) were categorized as intermediate risk and 10 (0.9%) as low risk at follow-up. For comparison, out of 9786 individuals (80.2% of total study sample) with low Lp(a) levels, only 256 (2.6%) and 21 (0.2%) moved to an intermediate and high risk category, respectively (*Figure 1C*). The majority of those that changed risk category had clinically relevant changes (≥25 nmol/L) across successive measurements (*Figure 1C*). In a complementary analysis, we investigated the stability of measurements that were deemed outside the analytical range (>189 nmol/L). Of the 1256 individuals with out-of-range values, 130 (10.4%) had values in the high risk range, 2 (0.2%) were categorized as intermediate risk, and 3 (0.2%) were categorized as low risk at the first follow-up visit.

## Discussion

Expanding on previous studies,^4^ we observed that while both low Lp(a) (<75 nmol/L) and high values (>189 nmol/L) remained stable over time, a significant proportion of individuals with measurements falling within these extremes (75–189 nmol/L) experienced clinically significant fluctuations in Lp(a) levels, resulting in changes in risk categorization. Specifically, almost one-third of those initially categorized within the intermediate risk range had Lp(a) levels in the high risk range at follow-up. Conversely, nearly one in five initially classified as high risk at baseline transitioned to the intermediate risk range upon subsequent assessment. Given the right-skewed distribution of Lp(a) in the general population, the majority of measurements are concentrated in the lower range. This disproportionate concentration may influence the overall correlation between the two measurements. By investigating the upper tail of the distribution, which includes individuals with values for which clinical guidelines are applicable, we were able to assess Lp(a) stability across the full range of values. These findings align with the observations from a recent ARIC study, which showed that particularly individuals with Lp(a) values in the intermediate risk range displayed large absolute changes.^5^ Additionally, large fluctuations were observed among individuals randomized to placebo in the IONIS-APO(a) Rx and IONIS-APO(a)-L Rx antisense oligonucleotide clinical trials.^6^ Our findings may have clinical implications. While a single measurement may suffice for those with Lp(a) < 75 nmol/L or >189 nmol/L, individuals with values between these extremes may benefit from repeat assessments to avoid misclassification. Furthermore, with the potential advent of novel Lp(a)-lowering therapies, accurate risk stratification and identification of treatment-eligible individuals become increasingly important.

## Declarations

### Data Availability

Data can be applied for through https://www.ukbiobank.ac.uk/enable-your-research/apply-for-access.

### Pre-registered Clinical Trial Number

None supplied.
